# Expression of micro-RNAs and genes related to angiogenesis in ccRCC and associations with tumor characteristics

**DOI:** 10.1186/s12894-017-0306-3

**Published:** 2017-12-04

**Authors:** Rita de Cássia Oliveira, Renato Fidelis Ivanovic, Katia Ramos Moreira Leite, Nayara Izabel Viana, Ruan César Aparecido Pimenta, José Pontes Junior, Vanessa Ribeiro Guimarães, Denis Reis Morais, Daniel Kanda Abe, Adriano João Nesrallah, Miguel Srougi, William Nahas, Sabrina Thalita Reis

**Affiliations:** 1 Laboratory of Medical Investigation (LIM55), Urology Department, University of Sao Paulo Medical School, Av. Dr. Arnaldo 455, 2° floor, room 2145, Sao Paulo, 01246-903 Brazil; 2 Uro-Oncology Group, Urology Department, University of Sao Paulo Medical School and Institute of Cancer Estate of Sao Paulo (ICESP), Sao Paulo, Brazil

**Keywords:** Angiogenesis, Genes, microRNAs, Renal cell carcinoma clear cell type

## Abstract

**Background:**

Clear cell renal cell carcinoma (ccRCC) is the third most common urological cancer in adults. Our aim is to evaluate genes and miRNAs expression profiles involved with angiogenesis and tumor characteristics in ccRCC.

**Methods:**

The expression levels of miRNAs miR-99a, 99b, 100; 199a; 106a; 106b; 29a; 29b; 29c; 126; 200a, 200b and their respective target genes: mTOR, HIF1-α, VHL, PDGF, VEGF, VEGFR1 and VEGFR2 were analyzed using qRT-PCR in tumor tissue samples from 56 patients with ccRCC. Five samples of benign renal tissue were utilized as control. The expression levels of miRNAs and genes were related to tumor size, Fuhrman nuclear grade and microvascular invasion.

**Results:**

miR99a was overexpressed in most samples and its target gene mTOR was underexpressed, this also occurs for miRNAs 106a, 106b, and their target gene VHL. An increase in miR-200b was correlated with high-risk tumors (*p* = 0.01) while miR-126 overexpression was associated with Fuhrman’s low grade (*p* = 0.03).

**Conclusions:**

Our results show that in ccRCC there are changes in miRNAs expression affecting gene expression that could be important in determining the aggressiveness of this lethal neoplasia.

## Background

Clear cell renal cell carcinoma (ccRCC) is the third most common urological cancer after prostate and bladder cancer and represents approximately 3% of cancers in adults as well as 85% of primary malignant kidney tumors [1]. Early detection and correct follow-up of the patients may influence the prognosis of the disease. Therefore, to improve our understanding of ccRCC pathogenesis, is necessary to identify new biomarkers enabling prediction of early metastasis after nephrectomy, and develop new targeted therapies [2].

MicroRNAs (miRNAs) are a class of small noncoding RNA with approximately 22 nucleotides and has critical roles in a many biological processes through translational repression or degradation of target messenger RNAs (mRNAs). Dysregulation of miRNAs has been shown to result in gene expression alteration and contributes to invasion and metastasis of many human tumors [3]. Prior studies indicated that miRNAs are also involved in other processes like tumor angiogenesis [4, 5]. Particularly, many urologic tumors have altered levels of numerous miRNAs [6]. Thus, changed expression of miRNAs may be useful for diagnosis, prediction of prognosis and treatment selection in patients with certain urologic cancers [7, 8]. Remarkably, targeted disruption of angiogenesis-related miRNAs may be a potential target treatment for RCC and other cancers [9].

ccRCC is characteristically highly vascularized and related to a germinative mutation of the von Hipple-Lindau (VHL) gene. Under adequate oxygen conditions, the VHL protein controls the expression of HIF-1α leading to its ubiquitination and degradation by the proteasome. In the absence of the VHL protein, due to gene mutation and or deletion, even during normal oxygen conditions HIF1-α will bind to the constitutively expressed HIF1, thus forming a heterodimer (HIF-1) activating transcription of genes related to angiogenesis and cell survival as VEGF, EGFR, PDGF, TGF-α, erythropoietin [10] ERK and mTOR. The discovery of the VHL-HIF1 pathway was responsible for the developing of target drugs represented by monoclonal antibodies and molecules responsible for tyrosine kinase inhibition action [11].

Some microRNAs have been called angiomirs because they regulate the angiogenesis process [12]. miR-126 [13]; miR-100, [14], miR-200 [15] and miR-26a [16] are some examples of this group of miRNAs.

Few studies have evaluated the presence of angiomirs in tumor development in urology, specially in ccRCC [17, 18]. Our aim is to evaluate genes and miRNAs expression profiles involved with angiogenesis and tumor characteristics in ccRCC.

### Clinical significance

The association between these biomarkers and ccRCC may contribute to the development of alternative tools that can facilitate the early detection and prognosis of this disease.

## Methods

### Patients

The study comprised the analysis of specimens from 56 patients diagnosed with ccRCC who were surgically treated by radical or partial nephrectomy at Hospital das Clinicas of University of Sao Paulo Medical School between January/2008 and March/2012 (Table 1). Final pathological status, tumor size, Fuhrman’s nuclear grade and micro vascular invasion were retrospectively reviewed. Patients were grouped according to a prognosis classification described by Dall’Oglio et al. into high, intermediate and low risk tumors [19]. The control group was composed of normal kidney tissues from patients with ureteropelvic junction obstruction.

**Table 1:** Pathological and Clinical characteristics of the patients

Age (years)	
Average	63.2
Median	51
Gender	
Male	37 (66.0%)
Female	19 (33.9%)
Parcial Nephrectomy	16 (28.6%)
Radical Nephrectomy	40 (71.4%)
Fuhrman’s Grade	
1–2	32 (57.2%)
3–4	24 (42.8%)
Microvascular Invasion	
Absent	44 (78.6%)
Present	12 (21.4%)
Pathological Stage (T)	
pT1-T2	26 (46.4%)
pT3-T4	30 (53.6%)
Pathological Stage (N)	
N0	54 (96.4%)
N1	02 (3.6%)
Pathological Stage (M)	
MO	48 (85.7%)
M1	08 (14.3%)

The expression levels of the following miRNAs (miR-99a, 99b, 100; 199th; 106a; 106b; 29a; 29b; 126; 200b) and their target genes (mTOR, HIF1-α, VHL, PDGF-β, VEGFA, VEGFR2) were correlated to ccRCC prognostic factors. Follow-up was 12 months at least and all samples had their confidentiality guaranteed.

Subjects provided written informed consent to participate the study and allowed their biological samples to be genetically analyzed. Approval for the study was given by the Institutional Board of Ethics (CAPPesq – Comissão de Ética para Análise de Projetos de Pesquisa) under the number 352891.

### Storage of samples

The surgical specimens were collected immediately after surgery. Tumor fragments with almost 1cm^2^ were placed into RNA later (Sigma-Aldrich, St. Louis, MO, USA) and stored at −80 °C. The samples were used for the analysis of miRNAs and target genes expression. We analyzed miR-99a, 99b, 100; 199th; 106a; 106b; 29a; 29b; 126 and 200b and their target genes mTOR, HIF1-α, VHL, PDGF-β, VEGFA and VEGFR2.

### miRNA isolation of RNA and cDNA synthesis

We used the mirVana kit (Ambion, Austin, TX) for RNA and miRNA extraction, and cDNA was obtained using the TaqMan miRNA Reverse Transcription kit (Applied Biosystems, Foster City, CA) for miRNA and the High-Capacity cDNA Reverse Transcription kit for RNA according to the manufacturer’s recommendations. A quantity of 10 ng miRNA was subjected to miRNAs of interest sequence-specific primer stem-loop reverse transcription. The PCR reaction for obtaining cDNA from miRNA was performed using Veriti equipment (Applied Biosystem, Foster City, CA) according to the following parameters: 30 min at 16 °C, 30 min at 42 °C, and 5 min at 85 °C. The same equipment was used to obtain cDNA from RNA with the following parameters: 10 min at 25 °C, 120 min at 37 °C, and 5 min at 85 °C.

### cDNA synthesis

Complementary DNA (cDNA) was obtained using miRNA TaqMan®miRNA Reverse Transcription Kit (Applied Biosystems, Foster City, CA) with the following quantities: A quantity of 200 ng/l miRNA was diluted in 20 μL of water. 3uL of this volume was subjected to reverse transcription, which was added 7 μL mix containing the kit reagents: 0,15 μl DNTP mix 0,5 μl the enzyme reverse transcriptase, the enzyme 1,5 μl buffer of 0,19 μl RNAse inhibitor, 3,66 μl nuclease of free water and 1 μl of primer with stem-loop sequence to specific miRNA totaling 10 μl cDNA. The PCR reaction for obtaining cDNA from miRNA was performed using Veriti equipment (Applied Biosystem, Foster City, CA) according to the following parameters: 30 min at 16 °C, 30 min at 42 °C, and 5 min at 85 °C.

The same equipment was used to obtain cDNA from RNA with the following parameters: 10 min at 25 °C, 120 min at 37 °C, and 5 min at 85 °C. The RNA cDNA synthesis was performed using the High-Capacity cDNA Reverse Transcription® kit (Applied Biosystems) using reverse transcriptase and random primers Multiscribe™. The total RNA was diluted in nuclease-free H2O to a final volume of 20 μl and concentration of 500 μL/​ng. In this volume were added 4 μl of random oligonucleotides (10X), 1,6 μl dNTP mix (25X), 4 μl of enzyme buffer (10X) 2 μl of the enzyme reverse transcriptase and 8,4 μl nuclease-free water. The solution was then subjected to temperature cycling (25 °C for 10 min, 37 °C for 120 min and 85 °C for 5 min) in Veriti® thermocycler (Applied Biosystems). At the end of both cDNA reactions were stored at −20 °C until use.

### Analysis of miRNA and RNA expression

The miRNA and RNA expression levels were analyzed by qRT-PCR using an ABI 7500 Fast Real-Time PCR System (Applied Biosystems). The target sequences were amplified in a 10 μl reaction containing 5 μl TaqMan Universal PCR Master Mix, 0.5 μl TaqMan Gene Expression Assays, 1 μl cDNA, and 3.5 μl DNase-free water. The PCR cycling conditions were 2 min at 50 °C, 10 min at 95 °C, and 40 cycles of 15 s at 95 °C and 1 min at 60 °C. All reactions were performed in duplicate, and TaqMan B2M and RNU 43 were utilized as the endogenous controls for gene and microRNA expression, respectively.

We used the CT method to calculate the relative expression of the microRNA and target genes using the formula CT = (CT target gene, ccRCC sample - CT endogenous control, ccRCC sample) – (CT target gene, Control sample - CT endogenous control, Control sample). The fold change in gene expression was calculated as 2^-CT^.

### Statistical analysis

Statistical analyzes were performed using SPSS 19.0 software for Windows (SPSS Inc., Chicago, USA). T student test or Mann-Whitney tests were used to compare the expression of miRNAs miR-99a, 99b, 100; 199th; 106a; 106b; 29a; 29b; 29c; 126; 200a, 200b with their target genes and ccRCC size, clinical stage and Fuhrman’s grade nuclear. A *p* value ≤0.05 was considered statistically significant for all calculations.

## Results

### ccRCC angiogenesis gene and miRNA expression profile

**Fig. 1:**
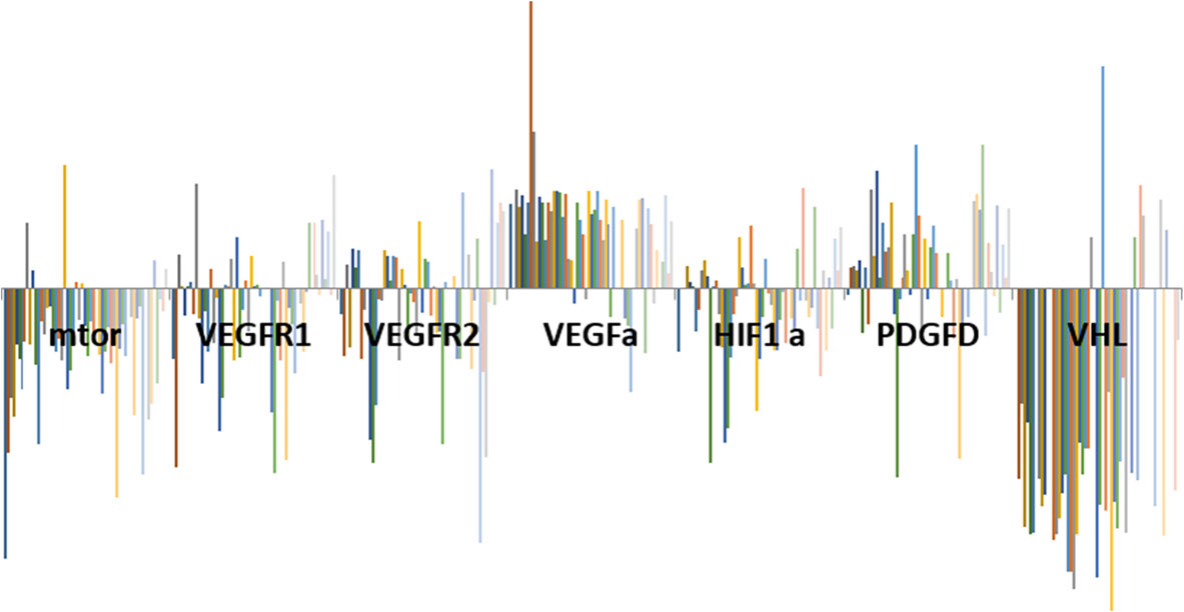
Graphical representation of the expression levels of genes related to angiogenesis in ccRCC tissue compared with control

**Fig. 2:**
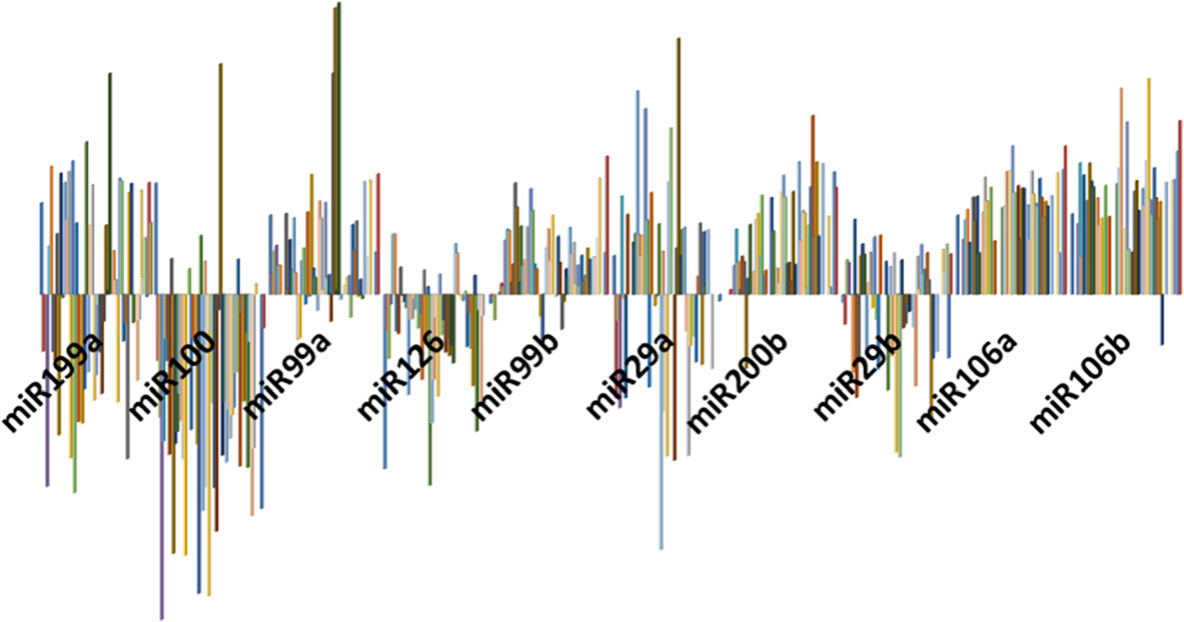
Graphical representation of the expression levels of miRNAs related to angiogenesis in ccRCC tissue compared with control

The genes with the highest overexpression were VEGFA (84%) and PDGF (86%) while mTOR (88%) and VHL (86%) presented the lowest expressions (Table 2). Genes with moderate expression included: VEGFR1 (59%), VGFR2 (55%) and HIF1-α (57%) (Fig. 1). The miRNAs which were overexpressed were: miR-99a (80.0%), miR-99b (94.0%), miR-200b (95%), miR-106a (100%) and miR-106b (97%). miRNAs 126 and 100 appeared underexpressed in 81.0% and 67.0% of patients, respectively. miRNAs 199a, 29a and 29b presented a very heterogeneous expression (Fig. 2).

**Table 2:** Gene expression and prognostic factors of ccRCC

	mTOR	VEGFR1	VEGFR2	VEGFA	HIF1A	PDGF	VHL
Low (*n* = 18)	0.63(1.32)	1.96(2.10)	3.17(6.12)	11.87 (16.14)	1.56 (2.27)	5.91 (11.18)	0.29 (1.04)
Intermediate (*n* = 29)	1.32 (5.43)	1.84(4.85)	1.11(1.38)	7.10 (4.47)	1.05 (1.29)	4.27 (9.39)	18.28 (87.65)
High *n* = 9	0.55 (0.63)	4.48(6.10)	1.51(2.26)	4.20 (4.58)	3.12 (5.58)	7.05 (9.53)	5.18 (6.73)
p	0.82^b^	0.37^b^	0.89^b^	0.18^b^	0.43^b^	0.76^b^	0.67^b^
Size ≥7 cm (*n* = 30)	0.52 (1.11)	1.74 (2.06)	2.41 (5.14)	10.33 (13,53)	1.27 (1.97)	6.62 (12.76)	0.39 (1.20)
Size <7 cm (*n* = 26)	1.54 (5.84)	2.69 (5.81)	1.31 (1.72)	6.32 (4.45)	1.74 (3.10)	3.56 (5.17)	18.84 (85.70)
p	0.97^a^	0.59^a^	0.87^a^	0.17^a^	0.50^a^	0.97^a^	0.12^a^
MVI absent (*n* = 44)	0.45 (0.95)	2.03(4.11)	2.08 (4.27)	9.77 (11.28)	1.10(1.63)	5.24(10.57)	12.54(72.60)
MVI present (*n* = 12)	3.39 (9.18)	2.75(4.76)	1.10 (1.71)	3.61(3.57)	2.91(4.36)	4.89(7.41)	3.66(5.73)
p	0.25^a^	0.61^a^	0.46^a^	0.57^a^	0.08^a^	0.91^a^	0.68^a^
Fuhrman’s Grade I e II (*n* = 32)	1.43 (5.32)	1.57 (1.79)	2.43 (4.97)	9.86 (13.09)	1.54 (2.11)	4.39 (9.21)	0.19 (0.84)
Fuhrman’s Grade III e IV (*n* = 24)	0.40 (0.52)	3.00 (6.10)	1.19 (1.68)	6.57 (4.81)	1.45 (3.06)	6.17 (10.97)	20.65 (89.28)
p	0.81^a^	0.45^a^	0.56^a^	0.26^a^	0.89^a^	0.51^a^	0.13^a^
Metastasis Absent (*n* = 48)	0.46(0.93)	1.81 (2.86)	1.96 (4.14)	8.53 (11.20)	1.54(2.76)	5.59 (10.78)	1.18 (3.44)
Metastasis Present (*n* = 8)	3.64(9.71)	4.03 (8.24)	1.41 (2.10)	7.98(4.54)	1.27(1.20)	3.05(3.04)	54.33(151.70)
p	0.82^a^	0.49^a^	0.71^a^	0.89^a^	0.77^a^	0.49^a^	0.79^a^
pT1–2 (*n* = 26)	0.51 (1.16)	1.33 (1.74)	2.47 (5.31)	10.12 (14.05)	1.47 (2.13)	7.07 (13.16)	0.20 (0.86)
pT3–4 (*n* = 30)	1.48 (5.62)	3.03 (5.63)	1.33 (1.72)	6.83 (4.67)	1.53 (2.95)	3.34 (4.95)	19.00 (85.67)
p	0.38^a^	0.51^a^	0.97^a^	0.26^a^	0.80^a^	0.16^a^	0.18^a^

^a^Mann-Whitney test ^b^ Kruskall-Wallis Test

### ccRCC angiogenesis gene and miRNA expression profile and prognostic factor

We studied the correlation between the ccRCC angiogenesis miRNAs and gene expressions with the prognostic factors. Considering gene expression, although not statistically significant we observed an increased of HIF1-α gene expression in patients with micro vascular invasion (*p* = 0.08). An increase in miR-200b correlated with high-risk tumors (*p* = 0.01) while miR-126 overexpression was associated with Fuhrman’s low grade (*p* = 0.03) (Tables 3 and 4).

**Table 3:** miRNA expression and prognostic factors of ccRCC

	miR-199a	miR-100	miR-99a	miR-99b	miR-126	miR-29a	miR-29b
Low (*n* = 18)	60.12(104.18)	0.51(1.34)	17.89(34.38)	22.01(37.5)	2.10(4.1)	19.25(28.23)	3.94(7.35)
Intermediate (*n* = 29)	59.99(159.22)	5.62(25.37)	22.18 (49.15)	13.07(31.1)	0.96(2.9)	10.90(28.49)	2.72(2.89)
High *n* = 9	48.64(85.19)	0.77(1.75)	44.44(75.01)	65.13(159.)	0.37(0.3)	7.05(9.72)	5.30(2.91)
P	0.65^b^	0.63^b^	0.50^b^	0.17^b^	0.29^b^	0.57^b^	0.47^b^
Size ≥7 cm (*n* = 30)	61.13(91.26)	5.69(26.31)	19.77(39.93)	22.79(42.3)	1.57(3.6)	13.80(24.88)	3.48(6.21)
Size <7 cm (*n* = 26)	55.80(166.90)	0.90(2.77)	27.68(56.40)	23.59(84.3)	1.02(2.1)	12.62(28.97)	3.29(2.90)
P	0.88^a^	0.36^a^	0.57^a^	0.96^a^	0.49^a^	0.88^a^	0.12^a^
MVI absent (*n* = 44)	63.00(145.83)	3.90(20.87)	15.77(34.2)	17.73(35.1)	1.55(3.2)	15.24(29.69)	3.19(5.13)
MVI present (*n* = 12)	43.28(73.27)	0.88(1.80)	51.97(76.99)	43.48(127.)	0.36(0.3)	5.37(7.45)	4.30(3.50)
P	0.65^a^	0.63^a^	0.05^a^	0.72^a^	0,43^a^	0.33^a^	0.54^a^
Fuhrman’s Grade I e II (*n* = 32)	39.95(87.23)	0.49(1.32)	23.61(45.13)	17.40(31.4)	1.92(3.7)	19.08(34.41)	3.63(6.04)
Fuhrman’s Grade III e IV (*n* = 24)	79.35(169.23)	6.35(26.88)	23.28(52.73)	29.41(88.9)	0.55(1.2)	5.70(7.80)	3.11(3.06)
p	0.28^a^	0.33^a^	0.98^a^	0.51^a^	**0.03** ^**a**^	0.10^a^	0,71^a^
pT1–2 (*n* = 26)	50.88(82.18)	6.36(27.40)	22.79(42.04)	23.35(43.8)	1.19(2.7)	14.85(25.19)	3.86(6.32)
pT3–4 (*n* = 30)	65.37(166.50)	0.69(2.59)	24.12(53.93)	23.01(81.1)	1.41(3.1)	11.84(28.52)	2.96(3.03)
P	0.69^a^	0.28^a^	0.92^a^	0.98^a^	0.78^a^	0.71^a^	0.51^a^
Metastasis Absent (*n* = 48)	49.76(84.62)	3.46(19.85)	19.01(39.85)	25.78(69.88)	1.17(2.54)	12.23(26.81)	3.61(418.33)
Metastasis Present (*n* = 8)	107.88(286.69)	1.89(4.94)	48.92(81.75)	6.41(6.35)	2.20(5.18)	18.95(28.74)	2.24(3.24)
p	0.25^a^	0.83^a^	0.12^a^	0.47^a^	0.39^a^	0.57^a^	0.46^a^

^a^Mann-Whitney test ^b^ Anova ^b^ Kruskall-Wallis Test

significant *p*-value are highlighted in bold

**Table 4:** Continuation of the miRNA expression and prognostic factors of ccRCC

	miR-200b	miR-106a	miR-106b
Low (*n* = 18)	43.39(89.17)	65.14(65.16)	159.44(171.94)
Intermediate (*n* = 29)	58.51(98.63)	110.15(141.57)	90.68(85.67)
High *n* = 9	563.01(1128.21)	202.94(270.20)	123.81(100.46)
P	**0.01** ^**b**^	0.23^b^	0.31^b^
Size ≥7 cm (*n* = 30)	57.91(97.01)	79.23(68.51)	125.34(140.61)
Size <7 cm (*n* = 26)	162.81(545.59)	139.51(196.72)	102.81(92.03)
P	0.36^a^	0.766	0.58^a^
MVI absent (*n* = 44)	55.97(95.09)	93.38(127.07)	119.47(126.44)
MVI present (*n* = 12)	355.35(899.73)	173.31(213.58)	94.63(90.02)
p	0.53^a^	0.17^a^	0.17^a^
Fuhrman’s Grade I e II (*n* = 32)	56.44(99.57)	103.91(158.18)	135.37(152.50)
Fuhrman’s Grade III e IV (*n* = 24)	169.48(557.25)	113.84(143.83)	101.01(92.55)
P	0.33^a^	0.83^a^	0.40^a^
pT1–2 (*n* = 26)	48.99(94.76)	67.86(60.49)	115.03(103.12)
pT3–4 (*n* = 30)	161.87(521.68)	143.33(187.99)	115.59(136.84)
P	0.33^a^	0.11^a^	0.98^a^
Metastasis Absent (*n* = 48)	119.99(0.47)	117.25(5.12)	109.83(122.37)
Metastasis Present (*n* = 8)	50.56(114.07)	54.21(43.87)	149.38(113.15)
p	0.66^a^	0.38^a^	0.50^a^

^a^Mann-Whitney test ^b^Anova ^b^ Kruskall-Wallis Test

significant *p*-value are highlighted in bold

Univariate analysis revealed that pathological stage, tumor size, Fuhrman’s nuclear grade and micro vascular invasion were significantly different in patients with high-risk tumors (*p* = 0.000; *p* = 0.000; *p* = 0.000; *p* = 0.000, respectively). To determine statistically significant clinical variables related to prognosis classification we used multivariate analysis to identify the importance of the isolated factors. Results showed that tumor size (0.475, 95% CI 0.436–0.777, *p* = 0.000), High Fuhrman’s nuclear grade (0.492, 95% CI 0.479–0.780, *p* = 0.000), micro vascular invasion present (0.332, 95% CI 0.378–0.730, *p* = 0.000) and miR-200b greater than 13.06 (vs 13.06 or more) (0.159, 95% CI 0.076–0.331, *p* = 0.003) were independently related to the high-risk tumors.

### ccRCC miRNA expression profile and target genes

A possible regulatory effect could be speculated for miR-99a overexpression (86%) and a mTOR underexpression (71.6%), A similar result was observed between VEGFA (77% overexpressed) and miR-126 (78% under expressed) and miR-106a (100%) and 106b (97%), both overexpressed; with VHL gene underexpression (86%) (Figs. 1 and 2).

## Discussion

ccRCC is considered the sixth leading cause of cancer deaths in Western countries. The incidence of this neoplasia is steadily increasing in the last years, each year; around 200,000 patients are diagnose with this disease resulting in approximately 100,000 deaths [20, 21]. The angiogenesis, process of development of capillaries from preexisting blood vessels, is essential for the growth of malignant tumors [22]. The VEGFA pathway plays an important role in regulating the process of angiogenesis, and von Hippel Lindau (VHL) gene loss or the hypoxia may explain the increased expression of this gene. In both situations, the HIF1-α gene moves to the cell nucleus, promoting VEGFA and PDGFa transcription [10, 23], As a result many drugs targeting angiogenesis have been developed and are currently being used to treat metastatic disease [24–26].

The importance of angiogenesis regulatory genes is not restricted to targeted therapy. Djordjevic et al. demonstrated that overexpression VEGFA gene was associated with high Fuhrman’s grade [27]. Also, Burgesser et al. correlated the expression of VEGFA, HIF1a, CD34 and Ki67 with most important prognostic factors in RCC in 83 patients. VEGFA expression was related to shorter survival rate after nephrectomy.

Our study is consistent with previous findings since we have found VEGFA overexpression that could be related to VHL under expression. It may be possible that in our cohort the mechanism involved in tumor development was VHL gene loss. However, we did not see HIF1-α overexpression in our cohort. On the contrary, HIF1-α was under expressed in 57% of our cases. However, when microvascular invasion was present, HIF1-α had higher expression compared to patients without microvascular invasion (no statistical significance).

Maybe, HIF1-α could be a marker of micro vascular invasion. Berghoffet. al. observed HIF1-α levels in brain metastasis samples from RCC, melanoma, breast, lung and colorectal cancer by immunohistochemistry. Increased HIF1-α levels were found in brain metastases from the RCC patients, and these had major effects on angiogenesis when compared to other tumors [28].

In the present study, a moderate PDGF gene expression (63%) could be seen. Wang et al. through microarray tests on 174 patients studied the role of PDGF-B and its receptor (PDGFR-β) in RCC progression. As PDGF-B expression increased among the samples a significant reduction in risk of death was found. In addition, PDGF-B induction in xenograft mice models could inhibit tumor growth and reduce cell proliferation [29].

In our study, mTOR was under expressed in 88% of patients, but it was increased in patients with adverse prognostic factors such as micro vascular invasion and metastatic disease, although not statistically significant. Similarly, Haddad et al. proved that mTOR, expression was inversely correlated to bad prognostic factors like high grade tumors and presence of necrosis in patients with ccRCC treated by nephrectomy [30].

Many papers reported changes in gene expression as prognostic markers. However they cannot be applied yet into clinical practice due to inaccuracy in comparison to known classical factors [31]. Thus, the investigation of new molecular markers cannot be considered finished.

At this point miRNA expression profile in ccRCC is not limited to determine new prognostic factors but also to explain molecular mechanisms related to its development and progression.

As previously mentioned, MicroRNAs are small noncoding RNAs that regulate the expression of target genes by translation repression or transcriptional regulation, so playing a role in many biological pathways [2, 7]. miRNAs have been classified as tumor suppressor or oncogenes [2]. Actually, miRNAs are estimated to regulate 30% of all gene transcripts, it is highly possible that their aberrant expression will contribute to ccRCC formation by altering the balance between oncogenes and tumor suppressor genes. When oncogenic miRNAs are overexpress in tumors, tumor suppressor genes are downregulated [6, 32].

Genes previously studied may be controlled by one or more microRNAs. miR-126 was under expressed in 78% of cases and has VEGFA gene as target that was overexpressed in 77% of the cases. Similar results were described by Khella et al. in a study where the opposite expression of miR-126 and VEGFA was identified [33]. In addition, in this paper, we provide important evidence in supporting of miR-126 functioning as a tumor suppressor in ccRCC, because miR-126 underexpression was associated with higher histological grade. miR-126 is considered a highly conserved miRNA with increased expression in vascularized tissues [34]. This miRNA has been reported to be associated with tumorigenesis of many types of cancer [35]. Wong et al. [36] showed that lower level of miR-126 represents tumor recurrence and poor survival in the HCC patients who received liver transplant surgery confirming our data in ccRCC.

VEGFA receptors can be controlled by miR-200b [37]. In our study, miR-200b was overexpressed while VEGFA receptors were moderately under expressed. While VEGFR1 and 2 genes were not correlated with any of the prognostic factors, miR-200b had a positive correlation with high risk neoplasms according to the pathological triad [19]. This is the first study to report miR-200b as a prognostic factor in ccRCC. Also, in prostate cancer our group demonstrated that overexpression of miR-200b correlated with localized tumors while its low expression was associated with locally advanced tumors (pT3), Gleason > 8 and shorter free biochemical recurrence survival [38].

Another inverse correlation, which suggests a regulatory mechanism is the VHL gene underexpression (86%) and its regulatory miRNAs overexpression: miR-106a (100%) and miR-106b (97%). The posttranscriptional inhibition performed by miR-106a and miR-106b may represent another mechanism other than gene mutation or even increase the effects of the mutation nullifying the intact allele. This is supported by experiments, which have silenced the VHL gene, leading to an increased in HIF1 and VEGFA expression [39]. These miRNAs have been described as prognostic factors in RCC but we were unable to demonstrate any association [40].

Finally, we demonstrated that miR-99a was overexpressed in 80% of the samples whereas mTOR was decreased in 88%. Cui et al. studied the effects of miR-99a over mTOR gene by miR-99a transfection in cell lines and RCC xenograft models. The miR-99a acts by inhibiting mTOR gene expression in both cell lines and in experimental models. It was confirmed that the high expression of miR-99a interfered with the G-1 phase of the cell cycle and delayed cell proliferation, decreasing tumor progression in vivo [41]. miR-99a did not correlate with clinical or pathological characteristics in our study, but the antagonist relationship with mTOR gene corroborates with the study by Cui et al. [41].

Some limitations of our study should be mentioned. This is a retrospective study and the number of patients is low in the control group and would be interesting to validate the results with a larger number of controls and cases enrolled prospectively. In a prospective study, it becomes easier to add behavioral characteristics of patients, like smoking and obesity that all may potentially interfere with analysis of recurrence, which was not possible in our work because we did not have this information. Moreover, the validation of these results in serum sample and functional studies in RCC cell line can improve the quality of our study and the clinical applicability of the results.

## Conclusion

In conclusion, we can postulate the role of miR-200b and miR-126 in the prognosis of this neoplasia. Our results support that in ccRCC there is an unbalance in the expression of genes and miRNAs related to process as angiogenesis, cell proliferation and survival. These finding besides helping us to understand the molecular mechanism of ccRCC gives us a reason to further investigate miR-126 and miR-200b as a potential biomarker and therapeutic target for this neoplasia.

## Abbreviations

ccRCC: Clear cell renal cell carcinoma
cDNA: Complementary DNA
miRNAs: MicroRNAs
mRNAs: Messenger RNAs
VHL: Von Hipple-Lindau
